# Exploring the Coating of Gold Nanoparticles with Lipids

**DOI:** 10.3390/nano15191516

**Published:** 2025-10-03

**Authors:** Mireia Vilar-Hernández, Jasper van Weerd, Pascal Jonkheijm

**Affiliations:** 1 Laboratory of Biointerface Chemistry, Department of Molecules and Materials, University of Twente, P.O. Box 217, 7500 AE Enschede, The Netherlands; m.vilarihernandez@utwente.nl; 2 LipoCoat BV, Hengelosestraat 541, 7521 AG Enschede, The Netherlands; jaspervanweerd@lipocoat.com

**Keywords:** gold nanoparticles, lipids, coating

## Abstract

(1) Background: gold nanoparticles (AuNPs) are of particular interest in biomedical research because they possess unique optical properties. In particular, its localized surface plasmon resonance is widely used for photothermal therapy and for detecting molecular interactions at nanoparticle surfaces. To enhance circulation time and biocompatibility, nanoparticles are often coated to shield their hydrophobic character. (2) Methods: we explored the seed-growth method to coat AuNPs with phospholipids to improve colloidal stability. (3) Results: various charged phospholipids were tested, and particle size and zeta potential were characterized. The monodispersity of the coated nanoparticles strongly depends on the narrow size distribution of both gold nanoparticles seeds and lipid vesicles. Achieving stable coated AuNPs with zwitterionic lipids such as phosphatidylcholine was challenging, whereas coatings containing phosphatidylglycerol did not compromise nanoparticle stability. (4) Conclusions: coating AuNPs with phospholipids via the seed-growth method has potential but requires further optimization to improve reproducibility and achieve stable nanoparticles with near-neutral surface charge.

## 1. Introduction

Metal nanoparticles such as gold nanoparticles (AuNPs) are of great interest for applications in, for example, medical diagnostics [1], imaging [2,3] and photothermal therapies [4] due to their unique optical properties. One of the most important characteristics is the localized surface plasmon resonance (LSPR). When a noble-metal particle is exposed to light, the photons of the electromagnetic field make the free electrons on the particles’ surface oscillate. When the frequency of both the electrons and the incident photons resonate, the LSPR leads to a strong absorption band [5,6]. The LSPR is characteristic of each metallic nanoparticle and will depend on size, shape and the environment [7,8,9,10,11]. LSPR is widely used to detect the binding of molecules to the nanoparticle surface, which causes a shift in the resonance wavelength [12,13].

To exploit the LSPR property of AuNPs, long circulation times, colloidal stability and antifouling properties are crucial for any therapeutic gold nanoparticle to reach the desired site in the body. However, due to the hydrophobic surface, AuNPs are prone to protein fouling and require stabilization to avoid aggregation and to maintain the colloidal solution. The interaction between the AuNPs’ surface and proteins can be controlled via surface modification. Generally, hydrophilic non-ionic polymers such as polyethylene glycol (PEG) derivatives are widely used to modify the AuNPs’ surface. PEGs reduce protein adsorption to the AuNPs and enhance the colloidal stability of AuNPs, because PEG provides a hydration layer covering the AuNPs’ surface and steric hindrance [14,15,16,17]. Other polymers that have been used to coat AuNPs for reducing protein deposition on the nanoparticle surface and preventing AuNP aggregation include hydrophilic cationic polymers such as chitosan [18]; amphiphilic non-ionic block copolymers such as polyethylene oxide-block-polypropylene oxide-block-polyethylene oxide (e.g., Pluronic F127 and P103) [19]; and different zwitterionic polymers [20] such as polybetaines.

In addition, different cell membranes containing molecules such as zwitterionic phospholipids have been studied to coat AuNPs with lipid (bi)layers [21,22]. Lipid (bi)layers are attractive for nanoparticle surface coating because they are biocompatible as they are composed of phospholipids naturally present in the cell [23]. Additionally, they exhibit antifouling properties [24], reducing non-specific adsorption of proteins on the nanoparticles’ surface in complex media for many medical applications [25]. This is attributed to the presence of zwitterionic phospholipids in the bilayer composition [26]. Moreover, they present high tuneability as their composition can be adjusted for specific applications by choosing from a wide range of commercially available lipids.

Different methods have been developed to enable the coating of AuNPs with phospholipids [27]. One of the simplest methods resulting in AuNPs with lipid bilayers uses the lipid film hydration method adopted from the synthesis of liposomes. This method starts with drying a lipid solution to form a thin lipid film, which is subsequently hydrated in an aqueous AuNP suspension and stirred or sonicated until a homogenous dispersion is achieved [22,28]. Another widely used method to assemble lipids on the AuNPs’ surface consists of two steps. First, an alkanethiol layer is assembled on the AuNP surface via sulfur–ligand (for example citrate) exchange. This alkanethiol layer serves as an anchor to facilitate the second step, which is the assembly of an outer lipid layer to the AuNP surface from rehydrated lipids present in the solution. In this lipid assembly method, a hybrid bilayer is formed where the inner leaflet binds covalently to the nanoparticle surface and the outer leaflet consists of phospholipids such as phosphoglycerol-based lipids (PG) or phosphocholine-based lipids (PC) [29,30,31].

A related method is the in situ synthesis of AuNPs in the presence of liposomes, typically involving cationic liposomes. After adding gold salt (HAuCl_4_) to a solution containing liposomes, a reducing agent is added slowly to initiate nanoparticle formation. During this process, vesicles adsorb onto the growing AuNP surface and form a lipid bilayer through vesicle fusion which stabilizes the AuNPs [32,33,34]. Alternatively, the synthesis of AuNPs inside liposomes has been explored. To this end, HAuCl_4_ is added to a solution of PC liposomes that encapsulate a reducing agent. Neutral gold ion complexes diffuse into the liposome, where they are reduced and grow into AuNPs, producing the PC-lipid-coated AuNPs [35]. When liposomes encapsulate both the gold salt and the reducing agent, by carefully warming up the solution, AuNPs can then be grown inside the liposomes to form bilayer-coated AuNPs [36]. Finally, a seed-growth method has been used to produce lipid-coated AuNPs. In this method, small AuNPs, which serve as nucleation sites, are mixed with liposomes. The gold precursor is then added, followed by the addition of the reducing agent, which leads to the growth of AuNPs inside the liposomes [37,38,39].

**Chart 1:**
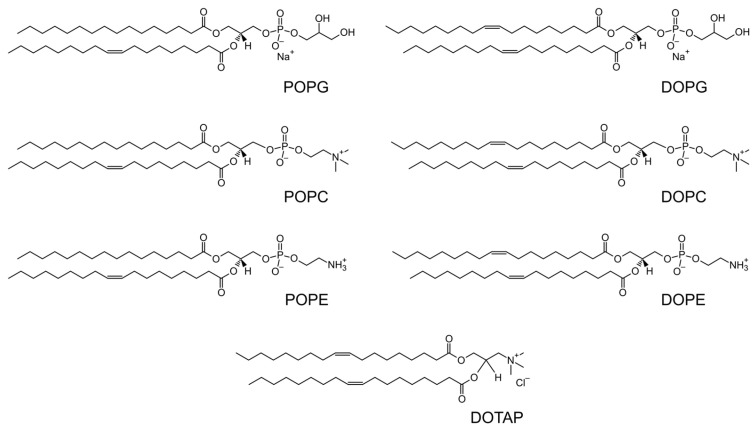
Chemical structures of the different lipids used in this study.

**Figure 1:**
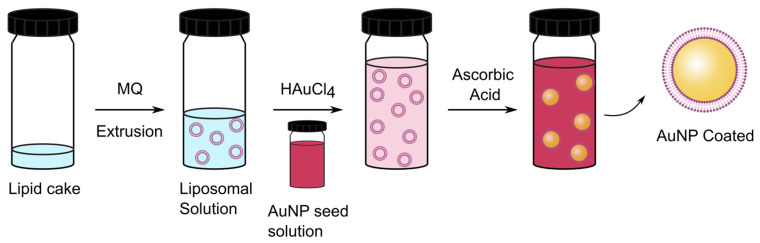
Synthetic scheme of coating AuNPs with lipids.

In this work, the seed-growth method was used to synthesize AuNPs with lipid coatings (Chart 1 and Figure 1) because it offers the best control over the size of AuNPs [40]. We employed different lipids for coating the AuNPs, such as anionic (PG), zwitterionic (PC, phosphoethanolamine based, PE) and cationic (trimethyl ammonium propane, TAP) phospholipids (Table 1). UV–Vis and DLS will be used to characterize the nanoparticles to assess their size and stability. The objective of this study is to identify which phospholipids can be used to form stable and effective coatings on AuNPs when employing the seed-growth method.

## 2. Materials and Methods

1-palmitoyl-2-oleoyl-glycero-3-phosphocholine (POPC), 1-palmitoyl-2-oleoyl-sn-glycero-3-phospho-(1′-rac-glycerol) (POPG), 1-palmitoyl-2-oleoyl-sn-glycero-3-phosphoethanol-amine (POPE), 1,2-dioleoyl-sn-glycero-3-phospho-(1′-rac-glycerol) (DOPG), 1,2-dioleoyl-sn-glycero-3-phosphocholine (DOPC), 1,2-dioleoyl-sn-glycero-3-phosphoethanolamine (DOPE), and 1,2-dioleoyl-3-trimethylammonium-propane (DOTAP) were purchased from Avanti Polar Lipids (Alabaster, AL, USA). Polycarbonate membranes of 50 nm were purchased from Whatman, (Cytiva, Marlborough, MA, USA). Sodium borohydride (NaBH_4_), gold (III) chloride (HAuCl_4_), sodium citrate (Na_3_Cit), ascorbic acid, chloroform 99%, nitric acid (HNO_3_) and hydrochloric acid (HCl) were purchased from Sigma Aldrich (Zwijndrecht, The Netherlands). The purchase of 5 nm gold nanospheres capped with 2 mM sodium citrate was from nanoComposix (San Diego, CA, USA).

Liposomes were prepared from chloroform stock lipid solutions. The appropriate amounts of the desired lipid were pipetted to achieve different molar ratios (Table 1). Then, the mixture was dried under a gentle nitrogen stream to create a thin film on the wall. The film was placed under vacuum for 1 h to completely remove the solvent. Next, the lipids were hydrated with Milli-Q water (Sigma Aldrich, EQ 7000, *ρ* > 18 MΩ cm^−1^) and vortexed for 1 min to create multilamellar vesicles. The solution was extruded 15 times through a 50 nm pore-size polycarbonate membrane, resulting in liposomes of around 90 nm at 1 mg/mL. The liposomes were stored under a nitrogen atmosphere at 4 °C for up to two weeks.

AuNPs were synthesized following the seed-growth method, adapted from M.S. Bakshi et al. [37]. All glassware was cleaned with aqua regia (4:1 (*v*/*v*) HCl (37%):HNO_3_ (65%)). A AuNP seed solution was prepared by adding Na_3_Cit to 25 mL of HAuCl_4_ (0.5 mM, final concentration) in water and stirred on ice for 10 min. Subsequently, 0.6 mL of NaBH_4_ (0.1 M, in cold water) were added and stirred for 5 min until a ruby-red color was observed, indicating the formation of AuNPs. Then, 0.5 mL of the AuNP seed solution was added to an aqueous (5 mL) solution containing liposomes (24 μg/mL) and HAuCl_4_ (0.5 mM, final concentration). After the addition of 0.2 mL (0.1 M) of freshly prepared ascorbic acid (AA), the solution was stirred for 30 min to allow the full reduction of the HAuCl_4_. All AuNPs were stored at 4 °C. This AuNP synthesis is schematized in Figure 1.

The hydrodynamic diameter, polydispersity index (PDI) and zeta potential of uncoated and coated AuNPs as well as the liposomes were measured using dynamic light scattering (DLS, Zetasizer Lab-Red, Malvern Panalytical, Almelo, The Netherlands). All measurements were performed in Milli-Q as triplicates. The UV–Vis spectra of coated and uncoated AuNPs were recorded at room temperature using a PerkinElmer (Shelton, CT, USA) Lambda 850 Ultraviolet–Visible Spectrophotometer (UV–Vis) in the wavelength range of 400–800 nm. Transmission electron microscopy (TEM) images were recorded after a droplet of nanoparticle solution was dried on a carbon foil with a Cu support grid. Images were recorded on an FEI-cubed titan Cs-corrected 80–300 kV TEM (Colorado Springs, CO, USA) with an initial low electron beam current to minimize possible beam damage to the lipid coating (0.5 e/Ås).

## 3. Results and Discussion

**Figure 2:**
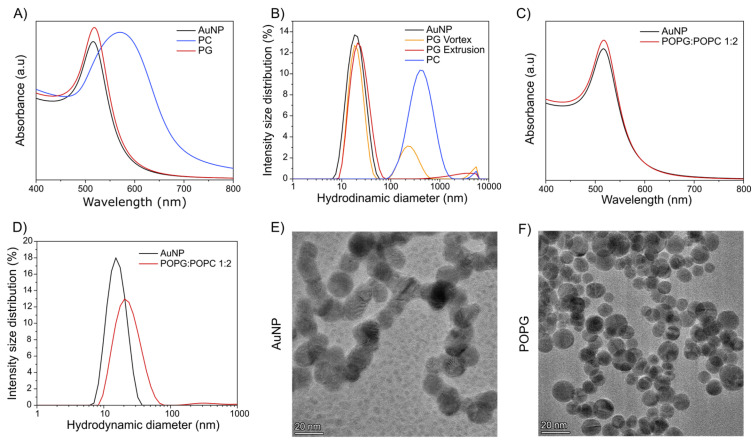
Characterization of AuNPs. (**A**) UV–Vis spectra of uncoated (black), PC- (blue) and PG- (red) coated AuNPs. (**B**) Intensity size distribution comparing uncoated (black) and PC- (blue) or PG- (red) coated AuNP prepared via the seed-growth method using vortexed rehydrated lipids (multilamellar vesicles) (orange) or extruded unilamellar vesicles (red). (**C**) UV–Vis spectra and (**D**) intensity size distribution of uncoated and POPG:POPC 1:2 coted AuNPs. TEM images of uncoated (**E**) and POPG-coated (**F**) AuNP.

AuNPs were synthesized following the seed-growth method, adapted from M.S. Bakshi et al. [37]. In brief, a freshly prepared AuNP seed solution was added to an aqueous solution containing multilamellar vesicles and HAuCl_4_ (Figure 1). It is particularly important to add the HAuCl_4_ before the AuNP seeds as it will affect the growth of the nanoparticles. After the addition of ascorbic acid and stirring for 30 min to allow the full reduction of the HAuCl_4_, the AuNPs were analyzed using UV–Vis spectroscopy, dynamic light scattering (DLS), zeta potential and transmission electron microscopy (TEM) (Figure 2). In agreement with the literature [37], coating AuNPs with POPC lipids resulted in particle clustering, as indicated by a large shift in the LSPR of AuNPs from a wavelength of 524 nm for uncoated AuNPs to 581 nm for POPC-coated AuNPs (Figure 2A). The coated AuNPs precipitated a few minutes after preparation.

In contrast, when PG lipid multilamellar vesicles were used, stable ruby-red colored suspensions were found after the addition of ascorbic acid, indicating proper AuNP formation [41]. The recorded UV–Vis spectrum in Figure 2A shows that the LSPR of the PG AuNPs was only slightly red-shifted and more intense compared to the uncoated AuNPs. This change in LSPR band is attributed to the increase in the local refractive index due to the lipid coating on the AuNPs [42]. This is in agreement with previous reports on lipid coatings of AuNPs when stronger interacting lipids are used during the seed-growth process. [37] The DLS analysis of these freshly PG-coated nanoparticles showed a bimodal intensity-weighted size distribution (Figure 2B, orange) with a peak at 21 nm, which could be related to the diameter of single AuNPs coated with lipids, and another peak with a size of *d* > 100 nm, which would most likely be due to lipids entrapping several AuNPs. To overcome this, the vortexed rehydrated lipids were extruded multiple times, which resulted in unilamellar vesicles of 90 nm diameter (Figure S1). When using these extruded lipid vesicles during the seed-growth method to coat the AuNPs, a monomodal size distribution was observed (Figure 2B, red) with an average diameter of 25 nm and a PDI of 0.2. Coating the AuNPs with extruded PG lipids led to an increase in the hydrodynamic diameter of 5 nm compared to uncoated AuNPs with a comparable PDI (Table S1). This data verifies the successful PG lipid bilayer coating on AuNPs without altering the stability of the nanoparticles. A solution with unilamellar vesicles improves the homogeneous coating on the AuNPs. This might be attributed to the increased efficiency of the rupture of smaller vesicles as well as the possibly more favorable size ratio of vesicles as compared to the generally smaller AuNPs [43,44]. When using seed solutions from commercial AuNPs with a more monodisperse size as compared to our AuNP suspensions, the same qualitative observations were made in the case of using PG, while a narrower size distribution was obtained (Figure S2 and Table S2). PG-coated particles remained stable for 10 days with no observed changes in the size distribution (Figure S3). Uncoated and PG-coated AuNPs were characterized using TEM (Figure 2E,F). Uncoated nanoparticles presented a pearl-necklace arrangement with fused borders. In contrast, PG-coated AuNPs showed a defined spherical shape; however, no visual presence of the coating was observed. It is possible that the coating prevented the fusion between the AuNPs.

**Figure 3:**
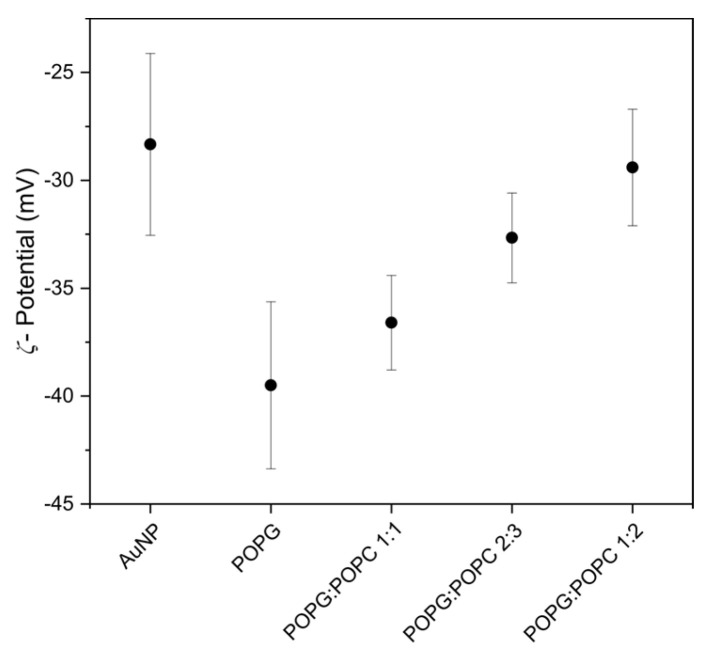
ζ-Potential of uncoated AuNPs and POPG, POPC at different molar ratios of coated AuNPs. Data represented as mean ± standard deviation for n = 3 measurements.

Next, zeta potential measurements were performed to evaluate the AuNPs’ surface charge, and the data is plotted in Figure 3. The addition of a PG coating on the AuNPs’ surface led to a 12 mV increase in negative charge than in the uncoated nanoparticles. To decrease the negative surface charge of the PG-coated AuNPs, as a next step, lipid films of mixtures of PG and PC were rehydrated, vortexed and extruded multiple times and then used to coat AuNPs during the seed-growth process. When increasing the fraction of PC in the PG vesicles, we observed that larger particles with higher PDI values in the AuNPs were made, but of monomodal size distribution (Table 2 and Table S3 and Figure 2C,D). When increasing the content of PC in the coating, the zeta potential increased accordingly (Figure 3), which further corroborates the successful coating of AuNPs with lipid bilayers of mixed PG–PC compositions. For PG-to-Pc ratios of 1:3, lipid-coated AuNP suspensions were aggregating within a few minutes after synthesis despite their initially promising particle sizes and PDIs. This observation aligns with the inability to synthesize a stable PC coating on AuNPs. It is likely that the zwitterionic character of PC interferes with the stability of the AuNPs and their interaction with the citrate. Instead, the negatively charged lipids, like PG, may act as a stabilizer, substituting the citrate. When the PC proportion remains below 60%, PG can still stabilize the AuNPs, while at higher PC fractions, the coated AuNPs become unstable. Therefore, 60% is the maximum PC content on a PG coating to have a stable AuNP suspension.

When other zwitterionic lipids such as PE or mixtures of PE and PG were used to coat AuNPs, a small shift in the LSPR band in UV–Vis spectra was observed when compared to the uncoated AuNPs (Table 2). When using a mixture of PE and PG in a 1:1 ratio to coat AuNPs, similar behavior was observed, as in the case of PG and PC lipids:

In order to achieve neutral surface charge of the PG-coated AuNPs, positively charged TAP lipids were added to the lipid mixture during the coating method (Table 3). TAP containing coating mixtures (with PC and/or PG) led to the observation of dark purple colors (LSPR band shifts 17–60 nm), which suggests the appearance of larger nanoparticles, as was confirmed using DLS measurements revealing large diameters with high PDIs. The small shift in the LSPR band in the case of PG and TAP in a 1:1 ratio (ruby-red color) is consistent with the previous results where the presence of the PG favors the coating of AuNPs. However, dispersions appeared to be unstable over time. To achieve a stable coating with near-AuNP-surface neutrality, which is generally believed to be advantageous for achieving antifouling performance, the addition of other stabilizers will be necessary.

## 4. Conclusions

The seed-growth method was explored for the preparation of lipid-bilayer-coated gold nanoparticles. A narrow size distribution for both the unilamellar lipid vesicle dispersions as well as the AuNP seed suspensions were found to be crucial for improved monodispersity of the product. For best antifouling performance, coatings based on only zwitterionic PC are preferred; however, the coating of AuNPs using only PC was more difficult to achieve using the seed-growth method as compared to negatively charged PG lipids, which are less optimal for achieving good antifouling performance. Charge compensation can be achieved by using PC in different molar ratios with PG to yield stable dispersions of lipid-coated AuNPs with average sizes of 30 nm to 60 nm. In the case of cationic coatings and their mixtures, the resulting nanoparticles are larger (100–200 nm).

Earlier studies using the seed-growth method showed that lipid bilayers could form on gold nanoparticles (AuNPs), but results were inconsistent. Coatings often relied on zwitterionic phosphatidylcholine (PC) or cationic lipids, which promoted vesicle fusion but frequently produced unstable or heterogeneous dispersions. In some cases, multiple AuNPs were entrapped within vesicles, leading to poor reproducibility. Our study builds on these findings by systematically testing a broader set of lipids, including anionic phosphatidylglycerol (PG), zwitterionic PC and PE and cationic TAP. We confirm earlier reports that PC alone yields unstable coatings and that TAP produces large, polydisperse particles. Importantly, we identify PG as a reliable stabilizer when unilamellar vesicles are used, yielding stable, monodisperse AuNPs of ∼25 nm. We also establish a quantitative threshold: PG must make up at least 40% in PG and PC mixtures to maintain stability. Overall, our work clarifies the role of lipid charge and vesicle size, refining earlier observations. However, further studies with other stabilizers would be needed to achieve stable dispersions of coated AuNPs with near-neutral surface charge, which is generally believed to have better antifouling properties.

## Figures and Tables

**Table 1:** Lipid molar ratios of PG, PC, PE and TAP headgroups. Compositions without symbols use only 1-palmitoyl-2-oleoyl tails.

Lipid	PC	PE
**PG**	1:0 ^1^	0:1 ^1^
	9:1 ^1^	9:1 ^1^
	1:1 ^1^	1:1
	2:3	
	1:2	
	1:3	
	0:1 ^1^	
	**PC**	**PG**
**DOTAP**	1:0 ^2^	
	1:1 ^2^	1:1 ^2^
	1:1:1 ^2^	

^1^ contains either 1-palmitoyl-2-oleoyl or 1,2-dioleoyl; ^2^ contains two 1,2-dioleoyl tails.

**Table 2:** Characterization of different POPG–POPC-coated AuNPs describing the LSPR band shift compared to the uncoated AuNPs, with the diameter based on the intensity peak and the polydispersity index (PDI). Data represented as mean ± standard deviation (UV–Vis) and median ± median absolute deviation (DLS) for n = 7 batches, each averaged for n = 3 measurements.

	Lipid Ratio	UV–Vis Shift	Diameter (nm)	PDI
**POPG:POPC**	1:0	1.8 ± 0.6	26 ± 1	0.20 ± 0.05
	1:1	1 ± 0.9	25.5 ± 5	0.36 ± 0.09
	2:3	1 ± 0.5	36.5 ± 13.5	0.35 ± 0.09
	1:2	1.3 ± 0.9	44 ± 20.5	0.36 ± 0.13
	0:1 *			

* not possible.

**Table 3:** Characterization of different DOTAP-mixtures-coated AuNPs describing the LSPR band shift compared to the uncoated AuNPs, with the diameter based on the intensity peak and the polydispersity index (PDI). Data represented as mean ± standard deviation (DLS) for n = 3 measurements.

	Lipid Ratio	UV–Vis Shift	Diameter (nm)	PDI
**DOPC:DOTAP**	0:1	48	136 ± 1	0.17 ± 0.02
**DOPC:DOTAP**	1:1	60	153 ± 7	0.41 ± 0.01
**DOPG:DOTAP**	1:1	0.5	162 ± 9	0.29 ± 0.02
**DOPC:DOPG:DOTAP**	1:1:1	17	211 ± 5	0.24 ± 0.01

## Data Availability

Data are contained within the article and Supplementary Materials.

## Supplementary Materials

The following supporting information can be downloaded at https://www.mdpi.com/article/10.3390/nano15191516/s1, Figure S1: DLS characterization of POPG extruded lipid vesicles vs. vortexed rehydrated lipids. On the left Intensity size distribution and on the right Z-average and polydispersity index (PDI); Figure S2: Intensity size distribution of POPG:POPC 1:2 coated AuNP using 5 nm AuNP Composixs as seed solution; Figure S3: Intensity size distribution of POPG coated AuNP after 10 days (red) stored in the fridge compared to day 1 (black) after the synthesis. Table S1: The LSPR band shift of uncoated vs. coated AuNPs and DLS measurements of POPG comparing the addition of vortexed rehydrated lipids (MLVs) versus extruded lipid vesicles (LUVs)j during the seed-growth method. Data represented as mean ± standard deviation (DLS) for n = 3 measurements; Table S2: Comparison of the DLS measurements of the coated AuNPs with the different synthesis method. For the own seed the data is same as Table 2 and for the Composixs data is represented mean ± standard deviation (DLS) for n = 3 measurements; Table S3: Characterization of different PG:PC and PG:PE coated AuNP describing the LSPR band shift compared to the uncoated AuNP, the diameter based on the intensity peak and the polydispersity index. Data represented as mean ± standard deviation (DLS) for n = 3 measurements.
