# Supplementary material for: Exploring the Coating of Gold Nanoparticles with Lipids
Source: Nanomaterials (Basel). 2025 Oct 3;15(19):1516. doi: 10.3390/nano15191516 (PMC12526019; doi:10.3390/nano15191516)
Supplement: Supplementary file 1 [file nanomaterials-15-01516-s001.zip › nanomaterials-3899203-supplementary.pdf]

Supporting information to Exploring the Coating of Gold Nanoparticles with Lipids – M.Vilar-Hernandez, J. van Weerd, P.Jonkheijm

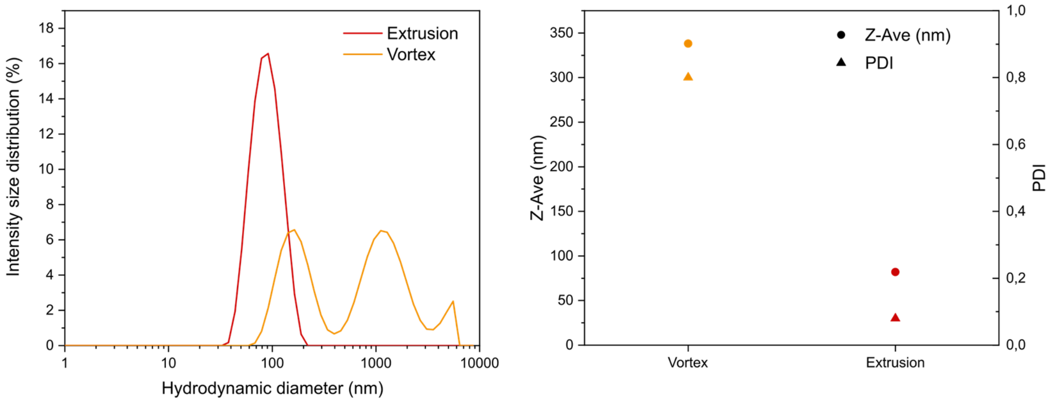

Figure S1. DLS characterization of POPG extruded lipid vesicles vs vortexed rehydrated lipids. On the left Intensity size distribution and on the right Z-average and polydispersity index (PDI).

Table S1. The LSPR band shift of uncoated vs. coated AuNPs and DLS measurements of POPG comparing the addition of vortexed rehydrated lipids (MLVs) versus extruded lipid vesicles (LUVs)j during the seed-growth method. Data represented as mean ± standard deviation (DLS) for n= 3 measurements.

	**UV-Vis shift**	**Diameter (nm)**	**PDI**
AuNP	0	21 ± 0.3	0.20 ± 0.01
POPG vortex	1	21 ± 0.6	0.42 ± 0.03
POPG Extrusion	1.8	25 ± 0.1	0.22 ± 0.01

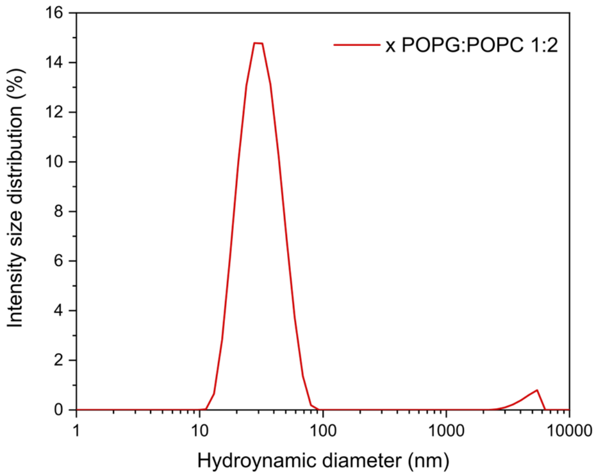

Figure S2. Intensity size distribution of POPG:POPC 1:2 coated AuNP using 5 nm AuNP Composixs as seed solution.

Table S2. Comparison of the DLS measurements of the coated AuNPs with the different synthesis method. For the own seed the data is same as **Table 2** and for the Composixs data is represented mean ± standard deviation (DLS) for n= 3 measurements.

	Own Seed		***Composixs***	
	**Diameter (nm)**	**PDI**	**Diameter (nm)**	**PDI**
AuNP	18 ± 3	0.21 ± 0.05	26 ± 1	0.16 ± 0.03
POPG	26 ± 1	0.20 ± 0.05	33 ± 3	0.21 ± 0.01
POPG:POPC 1:1	25.5 ± 5	0.36 ± 0.09	33 ± 1	0.18 ± 0.01
POPG:POPC 1:2	44 ± 20.5	0.36 ± 0.13	33 ± 1	0.21 ± 0.01

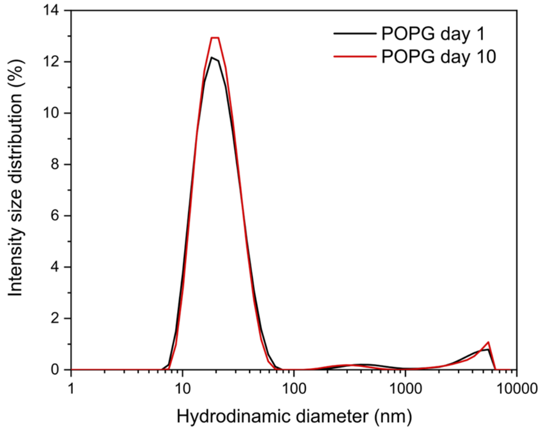

Figure S3. Intensity size distribution of POPG coated AuNP after 10 days (red) stored in the fridge compared to day 1 (black) after the synthesis.

Table S3. Characterization of different PG:PC and PG:PE coated AuNP describing the LSPR band shift compared to the uncoated AuNP, the diameter based on the intensity peak and the polydispersity index. Data represented as mean ± standard deviation (DLS) for n= 3 measurements

	DOPC				***DOPE***			
	**ratio**	**UV-Vis shift**	**Diameter (nm)**	**PDI**	**ratio**	**UV-Vis shift**	**Diameter (nm)**	**PDI**
DOPG	1:00	3.5	25 ± 1	0.23 ± 0.01	0:01	11	1188 ± 350	0.45 ± 0.05
	9:01	1	26 ± 1	0.28 ± 0.01	9:01	1	25 ± 1	0.26 ± 0.01
	1:01	1	27 ± 1	0.24 ± 0.01	1:01	1	25 ± 1	0.21 ± 0.01
	0:01	57	413 ± 34	0.26 ± 0.02				
	POPC				***POPE***			
POPG	9:01	1	26 ± 1	0.22 ± 0.01	0:01	-	-	-
	1:03	1	20	0.19 ± 0.02	9:01	1	23 ± 1	0.45 ± 0.02
	0:01	-	-	-	1:01	1	26 ± 1	0.25 ± 0.01
